# Lectures on Diseases of the Skin

**Published:** 1886-09

**Authors:** Henry Wile

**Affiliations:** Lecturer on Dermatology, Atlanta, Ga.


THE ATLANTA AND MEDICAL SURGICAL JOURNAL.

VOL. III. SEPTEMBER, 1886. NO. 7.

### Original Communications.

### LECTURES ON DISEASES OF THE SKIN.

DELIVERED AT THE ATLANTA MEDICAL COLLEGE DURING THE SESSION OF 1885–6, BY HENRY WILE, M. D., LECTURER ON DERMATOLOGY, ATLANTA, GA.

### LECTURE VII.

GENERAL TREATMENT—HISTORICAL—IMPORTANCE AND NECESSITY OF TREATMENT—EXTERNAL THERAPEUTICS—INTERNAL THERAPEUTICS—CLASSIFICATION.

Reported Stenographically for the ATLANTA MEDICAL AND SURGICAL JOURNAL by W. Kay Tewksbury.

Ancient medical observers regarded the skin as a channel whereby nature could throw off poisonous materials (materies morbi) circulating in the juices of the body, and diseases of the skin, supposed to be natural concomitants or results of the elimination of the poisons or humors, were therefore looked upon more as physiological processes. The process was supposed to continue as long as there was any poisonous matter in the system, so that a plan of treatment with a view to rapid cure was considered as dangerous; it was an interference with physiological functions, and disease of some internal organ was to be expected as a necessary consequence. The poisonous materials, instead of being allowed to escape, were "driven back" by treatment, and taking refuge in some internal organ, they were supposed to excite some, more or less, serious disorder. Cutaneous therapeutics, under the influence of such ideas, was practiced with cautious reserve. It was not until the beginning and middle of the present century, when all departments of medicine were revolutionized by discoveries, that dermatology took a new departure and threw off the fallacious theories that had determined its therapeutics for centuries. It was found to be expedient to treat diseases of the skin, and desirable to cure them, when possible, as quickly as it could be done. This has been firmly established by statistics and by the principles of modern comparative pathology. The skin being a vital organ of the economy, any serious disease that attacks it deserves the same prompt attention that disease of any other organ would command. Besides its own peculiar functions, the skin discharges certain complementary functions. Thus when the temperature of the body is suddenly raised by an increased heat production, as may occur from violent exercise or acute disease, it is brought to a normal condition by an increased activity of the sweat-glands. Likewise certain disturbances of the excretory function of the kidneys may be relieved by means of the cutaneous excretory apparatus. A normal condition of the skin is thus essential to health, and the proper discharge of its functions is most desirable in disease, especially of a febrile character. Chronic diseases, as chronic eczema rubrum and ulcerations attended with discharge which is rich in albumen, are more or less of a drain on the system, and, as in psoriasis, where the process of inflammation may involve large areas, there may be decided interference with a proper discharge of cutaneous functions. This, if long continued and neglected, must, in addition to the distressing subjective symptoms that accompany many diseases of the skin, seriously affect general health, besides being a constant source of worry and annoyance. Then, again, chronic diseases of the face, as acne atrophica, lupus erythematosus, besides being unsightly, are often followed by scarring, which is productive of disfiguration.

Still, notwithstanding the fact that the old theories as to treatment have been exposed by the results of modern investigation, they still have their adherents among general practitioners to-day, so firmly were they engrafted upon medical thought. It is not uncommon to meet a physician who would not only discourage attempts to heal a chronic ulceration, but even advise measures to favor its continuance. It is not unusual to meet those who treat cutaneous diseases under protest, and, with a consciousness of their own inability to carry out a rational course of treatment, will prescribe indifferently and empirically, regardless of the nature of the disease, its symptoms or stage of development, which are three important points to be kept in mind in treating this class of affections.

To treat diseases of the skin properly, the diagnosis must be correctly made, and the remedies must be adapted to the symptoms and stage of the process. In addition to this the effects of remedies employed should be known and their use regulated by the indications of each case. Where this is otherwise it is not to be wondered at that the treatment of diseases of the skin should yield unsatisfactory results, for in many instances the methods of treatment are of a hap-hazard character, and to transpose the idea of Voltaire, "they prescribe remedies, of which they know little, for diseases of which they know less."

Cutaneous therapeutics may be divided into external and internal. In some diseases external measures alone are required to bring about a cure, while in others a combination of external and internal medication is indicated. A successful treatment of cutaneous diseases bespeaks a recognition on the part of the physician of the indications in each case, whether for external or internal treatment, alone or combined. By external treatment the medicinal agents are brought into immediate contact with the diseased process, and effects are produced according to the nature of the agents and the method of their application. It must be remembered that the skin will often respond more quickly to stimulating applications in one individual than in another on account of the variation of the degree of sensibility. Thus where stimulation is necessary in two cases presenting the same symptoms, it may be necessary to make a number of applications more in the one case than in the other before the desired amount of stimulation is secured. In regard to this matter no definite rule can be prescribed. Each case presents its peculiarities that necessarily modify the method of treatment that is indicated.

The medicinal agents used in the external treatment of diseases of the skin are comparatively few in number, but the extent to which they can be manipulated and the variety of their combinations is almost endless. The remedies are employed in the form of liquids or lotions, as water, alcohol, glycerine, etc., pure or holding in solution or suspension solid substances as the salts of the metals; ointments or pastes with lard, lanolin, vaseline, paraffine or wax as a base; powders, solids in the form of caustics. They may be divided according to their properties, as *solvent*, *sedative astringent or absorbent*, *antiphlogistic*, *stimulating*, *disinfectant*, *anti-parasitic* and *caustic*.

As solvents, warm water alone or with soaps of various kinds; oils, as olive oil; fats, as lard, also vaseline and glycerine are used. Their purpose is to soften deposits of crusts and scales. Where the deposit is abundant the oils or fats used are allowed to remain for some hours until the desired effect is obtained. The surface may then be washed with warm water. Where the disease is extensive, covering large areas, the bath may be resorted to, and in such diseases as psoriasis and ichthyosis, in which the epidermis accumulates rapidly, it forms a valuable aid in the treatment. Baths may contain medicinal agents as bi-carbonate of soda, borax, or the various mineral spring waters may be used. They all exert but a macerating or softening effect on the epidermis. As cleansing agents, the soaps are servicable. Such as the common soft soap (sapo mollis) or green soap (sapo viridis), both potash soaps. The alcoholic solution of green soap, known as *spiritus saponatus kalinus*, composed of two parts of soap and one part of alcohol, filtered, is a most eligible detergent preparation.

Of the hard or soda soaps, the pure white castile is to be preferred.

As sedatives, astringents and absorbents there are the fats, oils and powders which are applied to protect affected surfaces from contact with the air, or to absorb secretions and discharges. For this purpose the fats, oils and vaseline are employed as ointments, alone or in combination with oxide of zinc, powdered starch, subnitrate of bismuth and litharge. These soft, emollient preparations are especially adapted to the treatment of acute processes; melting at the temperature of the body, they spread themselves evenly over the surface in the form of a thin, soothing, protecting layer. In addition to the powdered substances just given, a few others may be mentioned that are extensively used on account of their absorbent and astringent properties. These are Venetian talc, wheat flour, corn starch, lycopodium, powdered asbestos, carbonate of magnesium and powdered calamine.

Preparations containing some of the above powders, as oxide of zinc, calamine, and subnitrate of bismuth, suspended in water, as rose-water, cherry laurel water and orange flower water, may be prescribed with benefit as sedative astringent lotions.

As an antiphlogistic agent, warm and cold water, used in the form of a bath or applied by means of cold compresses, is often of great service in many acute and chronic diseases, as in erysipelas, phlegmonous inflammation, dermatitis and furunculosis. Applications of hot water as an adjuvant to the treatment of certain chronic inflammatory processes, as acne, rosacea, chronic eczema, are often productive of good results. Hot poultices of bread and milk, mush, or ground flax seed are indicated in processes attended with suppuration, as carbuncle, furuncle, abscess.

Remedies used for their stimulating properties are numerous, and one must be governed in the choice of them by the amount of stimulation required by the diseased process, and also by the special properties of these agents, as some of them are peculiarly effective under certain conditions. These conditions will be mentioned in subsequent chapters. To be able, therefore, to select appropriate remedies, a comparative knowledge of their special properties is requisite. They are usually prescribed in the form of ointments or lotions, or sometimes in combination with gelatine, collodion and guttapercha, and the degree of stimulation can be regulated by the amount of the remedy used. Among these agents the most valuable are as follows: mercury and its preparations, as calomel, corrosive sublimate, white precipitate, red oxide, oleate and ointment of the nitrate of mercury called citrine ointment; sublimed and precipitated sulphur, boracic acid, benzoic acid, chrysophanic acid or chrysarobin, resorcin, salicylic acid, veratria, naphthol and ichthyol. The different tarry preparations, as pix liquida, oleum cadini (juniper), oleum rusci (birch), oleum fagi (beech), tincture rusci an alcoholic preparation of the oil, liquor carbonis detergens an alcoholic solution of coal tar; spiritus saponatus kalinus. These various remedies are also incorporated in a certain percentage with soaps; thus sulphur soap, salicylic acid soap, naphthol soap, etc. But the objection urged against the employment of these soaps as curative agents lies in the fact that the quantity of the remedy used and the amount of stimulation produced in each application cannot be determined.

Disinfectants are useful at times, especially in the treatment of chronic ulcerations, as epithelioma and late syphilitic ulcerations. The ones most generally preferred are permanganate of potash, boracic acid and corrosive sublimate. They are used in solutions of varying strength. The medicated soaps just mentioned may here be used to good advantage.

As anti-parasitics there are a number of remedies, so called on account of certain properties that render them fatal to parasitic organisms whose presence and development in the layers of the skin occasion disease. Against vegetal parasites the preparations of sulphur and mercury are most efficacious. Of the former precipitated sulphur, sulphite and hyposulphite of soda, sulphurous acid; of the latter corrosive sublimate, ammoniated mercury, red iodide, acid nitrate in form of citrine ointment are the most important. Besides these, carbolic acid, creosote, iodine and acetic acid may be used with good results. They are used, as a rule, in combination in the form of ointments or lotions. Against animal parasites the most reliable preparations are petroleum, ointment of mercury, staphisagria (seeds), cocculus indicus (kernels), naphthol, Peruvian balsam and sulphur.

Caustics are used to destroy diseased tissue, to make a profound impression upon or to excite an acute inflammation at the seat of a chronic process. The most powerful of these are the strong mineral acids, as fuming nitric, sulphuric, hydrochloric, and the alkalies as potassa, soda, lime. In addition to these are others that are often used, as nitrate of silver, chloride of zinc, chloride of antimony, arsenious acid, chromic acid and carbolic acid. The caustic potash and nitrate of silver are applied, as a rule, alone. The other agents are used more in the form of pastes, of which there are many, thus:

Vienna paste, caustic potash and lime, equal parts mixed with alcohol.

Canquoin's paste, chloride of zinc, one part deliquesced, three parts of powdered starch.

Landolfi's paste (modified), chloride of zinc two parts, chloride of antimony two parts, concentrated hydrochloric acid one part, powdered licorice q. s. to make a paste.

Arsenic paste (Hebra), arsenious acid one part, cinnabar three parts, vaseline twenty-four parts.

Besides the use of chemicals there are two other methods of obtaining a caustic action. Thus the galvano and thermo (Paquelin) cautery are of special value, in that the effect of the agent can be exactly measured; it is under control more completely than chemicals, and little time is consumed and much less pain occasioned in securing the result.

In the external treatment of diseases of the skin certain operative measures still deserve mention. These are scarification to reduce chronic hyperæmia, the use of the curette or scraping spoon to remove morbid products ; the electrolysis needle for removing superfluous hairs, also obliterating blood vessels to reduce chronic hyperæmia, and the application of electric currents, both galvanic and faradic, in certain diseases attended with nerve disturbance.

Internal cutaneous therapeutics offers but few remedies that exert decided influence upon affections of the skin. The most important of these are preparations of mercury and the iodides of potassium and sodium used especially in the management of syphilitic eruptions, and arsenic in the form of asenious acid or Fowler's solution, employed more especially in chronic inflammatory processes, as chronic eczema and psoriasis.

The effect of mercurials and the iodides on syphilitic diseases is, as a rule, prompt and efficient; whereas the action of arsenic is slow and its use must be continued for a time after the disease disappears. Arsenic has from time immemorial enjoyed the reputation of being a panacea for all cutaneous ailments. It was prescribed indiscriminately without plan or purpose, and the results were accordingly good, bad and indifferent, mostly the last. It has been shown that arsenic has a decided stimulant action upon the nutrition of the mucous layer of the epidermis and to use it in diseases in which there is already too much stimulation would only aggravate the process. The use of this drug is being more and more limited, and experience with careful observation has shown that it has no appreciable effect in the great majority of diseases for which it is ordinarily prescribed. In chronic eczema and psoriasis it is specially recommended, but it is only in particular stages of these diseases that any good effect may be expected. The remedy is to be administered after meals, and when in liquid form, well diluted with water. It should be prescribed with caution and toxic effects avoided. There are many other remedies that are used as tonics, aperients, diuretics and agents generally to correct disorders of the blood and digestive system. The most important are the preparations of iron, quinine, codliver oil, sulphate of soda, sulphate of magnesium, mineral waters, alkaline diuretics, opium, belladonna, ergot and astringents as nitrate of silver.

In the treatment of certain diseases, particularly those that bear relations to disorders of the alimentary canal, relations especially of a reflex character, the regulation of the diet is an essential element. It will be found that indulgence on the part of the patient in certain articles of food, as shellfish, strawberries in urticaria or indigestible food in acne and eczema, may aggravate the disease or bring about a relapse.

Finally, in regard to internal treatment, it may be added that one must be governed, in most cases, by the general condition of the patient, by the extent to which the cutaneous affection is related to some internal disorder, and by the nature of that internal disorder. Just here is one of the intimate connections of dermatology with general medicine. A knowledge of dermato-therapeutics includes a knowledge of the principles of general therapeutics, and to meet successfully the requirements of each case the latter must act as a guide.

Before concluding the subject of general dermato-pathology, of which the lectures have thus far treated, I wish to remark briefly on the question of classification. The classification of diseases of the skin has engaged the attention of such writers as Galen, Mercurialis, Lorry and Shönlein, but the first systematic work in this direction was done by Plenck (1776), of Vienna, followed by Willan and Bateman (1798), of England, and Cazenave and Gibert, of France. According to their system diseases were classified with regard to symptoms or morbid products.

Later Erasmus Wilson, of England, classified the diseases according to their anatomical position, as those of the epidermis, hair follicle, nerve, blood-vessel, etc.

Bærensprung, of Germany, made three divisions: I. Disorders of innervation; II. Disorders of secretion; III. Disorders of nutrition.

Hebra's classification, based mainly upon the pathologico-anatomical characters of the diseases, is one adopted by most writers on dermatology of the present day. It is the most scientific, simplest and practical system yet proposed, and with the modifications that have been made from time to time to meet the advance made in our knowledge of these diseases, it meets the requirements better than any system yet proposed.

Auspitz, of Vienna, recently (1881) proposed a new classification composed of nine classes, each divided into families with further subdivisions. It is based upon a clinical unity of diseases and upon the general pathology of diseases when the latter coincides with the clinical characters of the diseases. This system is too theoretical, and for practical use entirely too complicated.

It will be my purpose in the remaining lectures, which will treat of the special pathology of diseases of the skin, to follow the classification of Hebra as modified by Duhring, thus:

Class I. Disorders of secretions embracing diseases of the glandular apparatus.
" II. Hyperæmias.
" III. Inflammations.
" IV. Hemorrhages.
" V. Hypertrophies.
" VI. Atrophies.
" VII. New formations.
" VIII. Neuroses.
" IX. Parasites.

These classes are further subdivided according to the special anatomical, clinical and morphological characters of the diseases which they embrace.

